# The Structural Integrity and Fracture Behaviour of Teeth Restored with PEEK and Lithium-Disilicate Glass Ceramic Crowns

**DOI:** 10.3390/polym14051001

**Published:** 2022-03-01

**Authors:** Sami Aldhuwayhi, Muhammad Syafiq Alauddin, Nicolas Martin

**Affiliations:** 1 Department of Prosthodontics, College of Dentistry, Majmaah University, Al-Majmaah 11952, Saudi Arabia; s.aladdowihi@mu.edu.sa; 2 Department of Conservative Dentistry & Prosthodontics, Faculty of Dentistry, USIM, Kuala Lumpur 55100, Malaysia; 3 Academic Unit of Restorative Dentistry, University of Sheffield, Sheffield S10 2TA, UK

**Keywords:** PEEK, IPS e.max^®^CAD, structural integrity, fatigue life and limit, dental crown

## Abstract

Polyetheretherketone (PEEK) is a unique polymer material which has recently been introduced to dentistry. This study aimed to assess the structural integrity of PEEK as a posterior tooth crown and compared it with ceramic-based material. A total of 31 monolithic CAD-CAM PEEK (Juvora^TM^, Strumann, Andover, MA, USA) crowns and 31 lithium disilicate (IPS e.max^®^CAD, Voclar Vivadent AG, Liechtenstein) crowns were created and cemented on dentin-like teeth (AlphaDie^®^MF, Schütz Dental GmbH, Rosbach, Germany) in a precise procedure mimicking the physical and mechanical properties of natural teeth and periodontal ligaments. A static compressive strength test using a universal testing machine and a dynamic fatigue test using a chewing simulator machine were used until crown failure to assess the fracture behaviour by mode of fracture (fractographic analysis) and 3D digital subtraction analysis. The results showed that PEEK has a greater fracture resistance than IPS e.max^®^CAD by 2060 N to 703 N. Additionally, in fatigue limit, IPS e.max^®^CAD showed a constant failure under 2.0 Kg (=320 N) before 5000 chewing cycles while PEEK survived at a significantly higher load > 11 Kg (930 N). Furthermore, PEEK showed a continued survival at >1,250,000 cycles while the mean fatigue life of IPS e.max^®^CAD was around 133,470 cycles. PEEK illustrated a significantly less catastrophic failure mode with some plastic deformation at the fractographic stereomicroscope and in the 3D digital subtraction analysis. Using PEEK for crowns looks very promising, however, further clinical studies are required to assure this study’s results.

## 1. Introduction

A wide range of materials can fabricate full-coverage crowns for the restoration of structurally compromised teeth. Metals, metal ceramics and all ceramic crowns are the most prevalent materials with good survival rates and clinical properties (mechanical and aesthetic) derived from the inherent characteristics of the material [1,2,3,4,5,6,7]. To date, polymer-based materials have been characterised by a low fracture strength and wear rate which has made them unsuitable for this restorative purpose. A new group of such materials have been developed in recent years from the generic group of polyaryletherketone (PAEK). Within this group, the material polyetheretherketone (PEEK), a semicrystallize thermosoftening polymer, has the potential for a framework for fixed and removable prostheses by virtue of its biocompatibility and desirable physical and mechanical properties [8,9]. PEEK materials have rigidity comparable to bone fixation plates for fractured bones, orthopaedic implants, joint arthroplasty, and vertebral cages [10,11]. In dentistry, PEEK has various applications due to its good biological, mechanical, aesthetic, and handling properties with excellent results in comparison to other conventional materials [12,13,14,15,16,17,18,19,20,21].

The use of PEEK for extra-coronal restorations with a laminated outer RBC is a new application that requires testing and validation. In addition to assessing the material’s inherent properties per se, it is equally important to determine its structural integrity as part of a tooth-restoration complex. This can be achieved through mechanical stress tests in the form of monotonic (static test—fracture test) or cyclic (dynamic-fatigue cyclic loading) tests [22,23]. Fatigue can be defined as the strength degradation and weakening of a material caused by repeatedly applying loads which ultimately could cause material failure [2]. Fatigue stress due to repetitive occlusal load has been proven to be the most common reason for clinical dental prosthesis failure [24,25]. The fatigue behaviour of any material structure is achieved by measuring the fatigue limit and the fatigue life of the test specimen. Several studies have used both concepts [15,17,18,21,24,26]. Fatigue limit is the maximum amount of stress that the structure can survive for a predetermined number of loading cycles. The most suitable method to be evaluated used the staircase technique and the average number of samples used were between 10 and 20 [15,17,18]. One the other hand, fatigue life is the maximum number of cycles that the body can withstand without failure for the given amount of stress. Survival of more than 1,200,000 chewing cycles (representing five years of clinical service) is considered clinically acceptable [19,20]. The number of samples needed for fatigue life test varies between the studies depending on testing procedure, the higher limit of fatigue life, the machine, and material used etc. Variable fatigue testing and chewing simulating machines have been used in fatigue test studies [13,15,18,19,24,27,28]. Comparing the outcome data between these different machines is impossible as they depend entirely on the testing machine. Notwithstanding this in vitro simulation, mechanical setup allows for performance comparison of materials and/or restorative systems that are subject to the same loads. One of these fatigue testing machines has been constructed by Mair et al. and validated in several studies [15,16,29]. This mechanical machine consists of five independent stations with the specimens submerged in a controlled temperature water bath. Test specimens are subjected to the impact of indenters of a specific form and shape (representing an antagonist molar cusp).

A challenge with in vitro testing of restorative systems arises in the choice of dental substrate. Natural teeth are more realistic and represent the actual crown substructure in the mouth. Their use in these studies is limited by their availability and the significant anatomic, histological, and structural variability. Polymer replica teeth with matched elastic modulus close to dentine provide a standardised substrate for the tests within and across groups [20,21,30,31,32].

This study aims to undertake a comparative assessment of the crown-tooth complex’s structural integrity and fracture behaviour when restored with either PEEK or lithium disilicate glass ceramic crowns that are adhesively cemented to premolar polymer typodonts. The results will help predict the clinical performance, durability, and failure mode of machined monolithic PEEK crown on posterior teeth.

## 2. Material and Method

A lack of data from previous literature that directly compared lithium disilicate glass ceramic IPS e.max^®^CAD (control group) to machined PEEK crown (test group) made it difficult to develop a power calculation. The test was repeated in a sequential manner until a statistical significance of <0.05 (*p* < 0.05) was achieved. Due to the identical and carefully controlled samples, the approach in this study was to undertake an initial investigation using 10 samples of each group for static testing and 30 samples of each group were prepared for both dynamic tests following the chewing simulator machine designer’s recommendations.

### 2.1. Tooth Preparation

A total of two identical typodont upper right second premolars (AG-3, Frasaco, Frasaco GmBh, Tettnang, Germany) were prepared to receive a full coverage Monolithic PEEK crown (Juvora^TM^) and a monolithic lithium disilicate (IPS e.max^®^CAD, Ivoclar Vivadent, Schaan, Liechtenstein) crown in accordance with the required preparation guidelines (Table 1). Consistency of preparations was achieved through the use of a parallelometer to hold a dental turbine and a bur at the required taper angulation. 

### 2.2. Tooth Duplication, Mounting and Alignment

**Figure 1:**
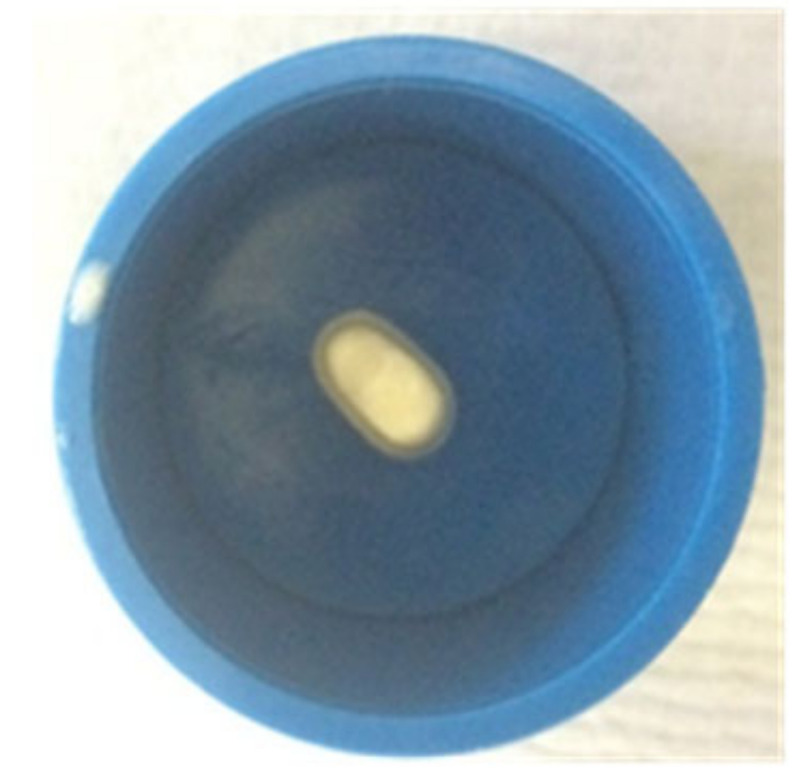
The tooth stacked in the centre of plastic cylindrical rings.

**Figure 2:**
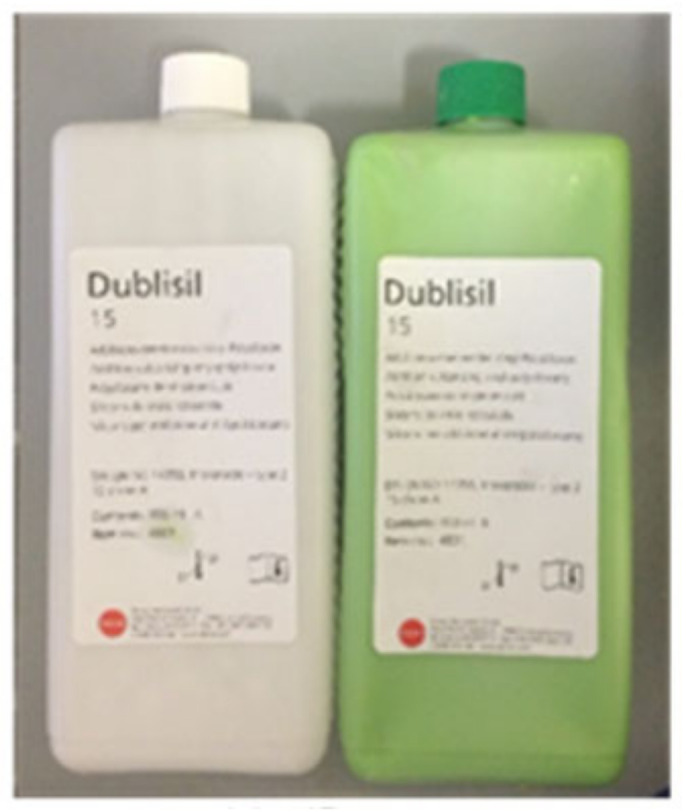
Dublisil^®^ 15.

**Figure 3:**
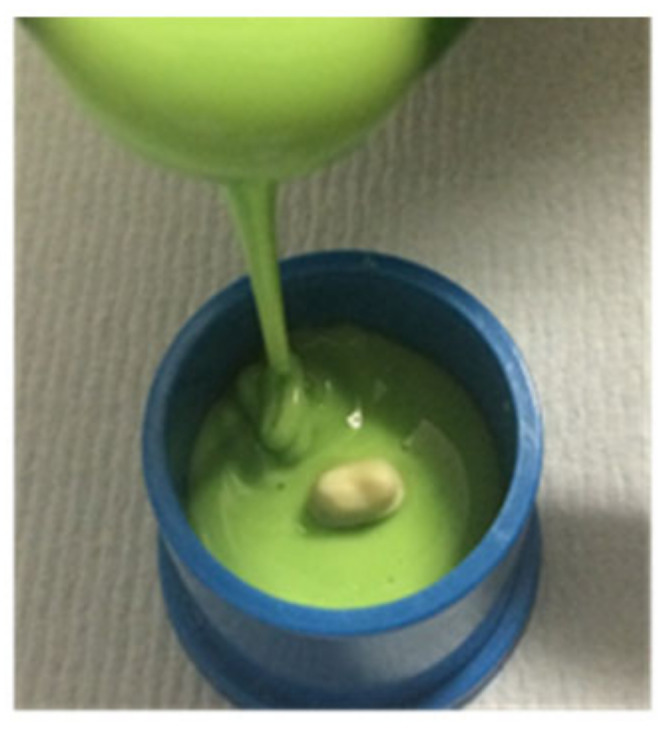
Gently pour the impression.

**Figure 4:**
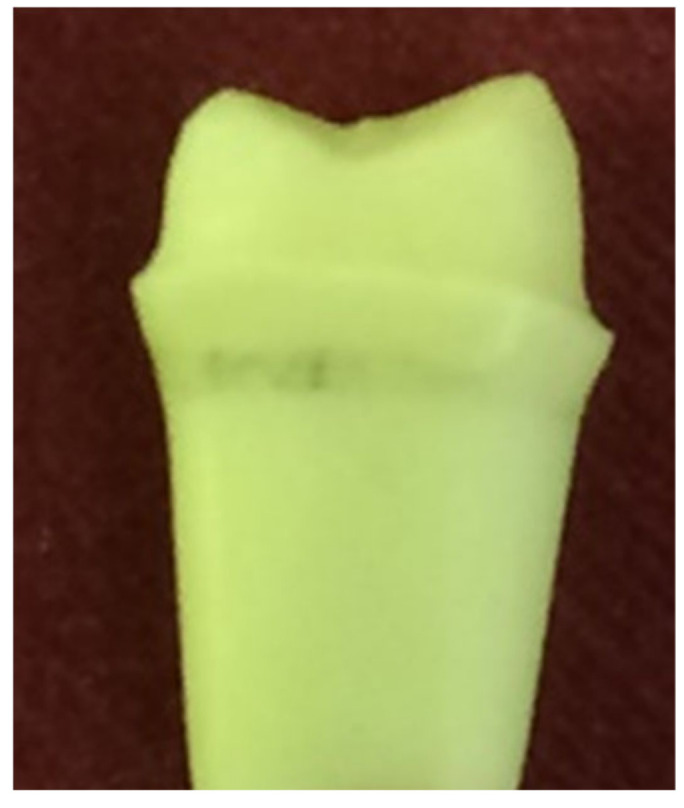
Original Frasaco prepared teeth of each group were marked 2 mm apical to the cemento-enamel junction circumferentially.

**Figure 5:**
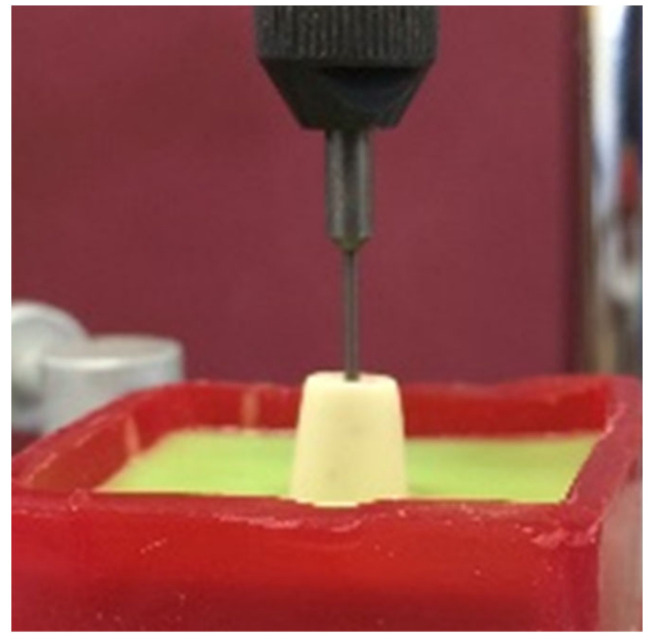
Teeth were then separately placed and stacked in an upside down vertical direction following their long axis with the help of a dental surveyor pin inserted in the typodont tooth screw slot.

**Figure 6:**
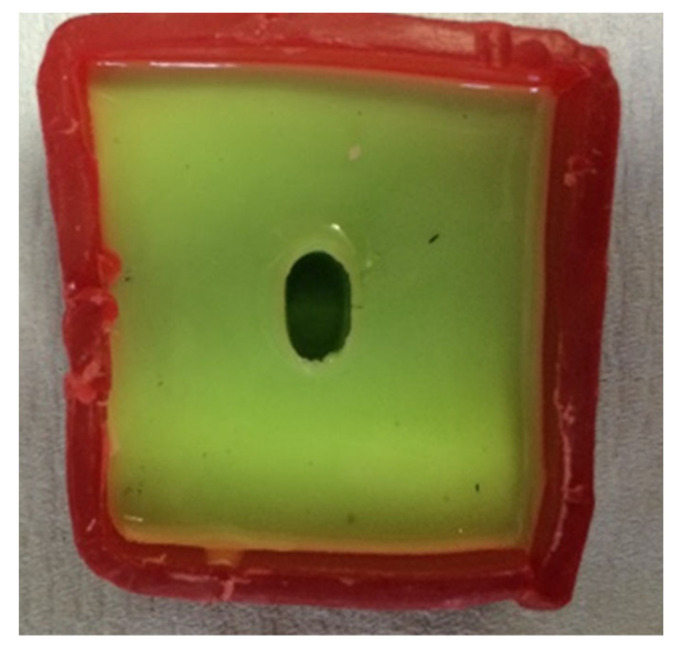
A wax boarded box container mounted on a 0° degree tilted articulator table was filled with freshly mixed addition silicon impression material (Dublisil^®^ 15) to embed the prepared tooth until the marked line. This procedure was applied to both original prepared typodent teeth to fabricate 2 original moulds of the ideal teeth alignment of both groups which was used for the rest of the samples.

**Figure 7:**
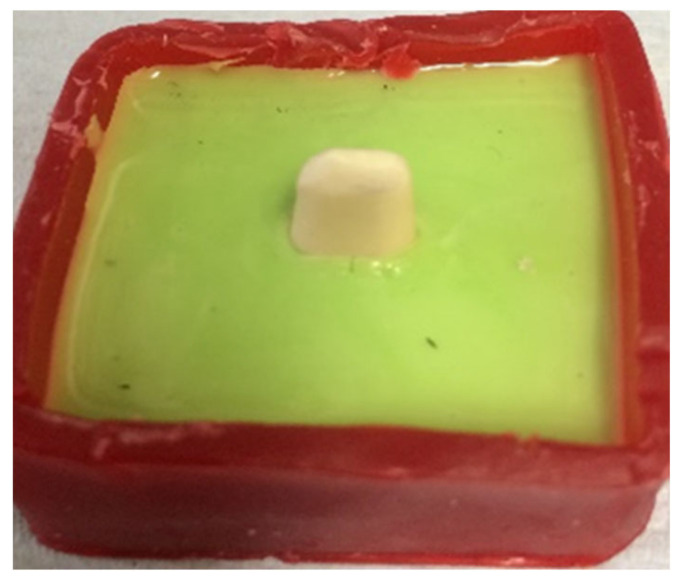
The duplicated samples then were separately painted with a thin layer of die separator spray and their coronal part was inserted in the alignment mould of their group.

**Figure 8:**
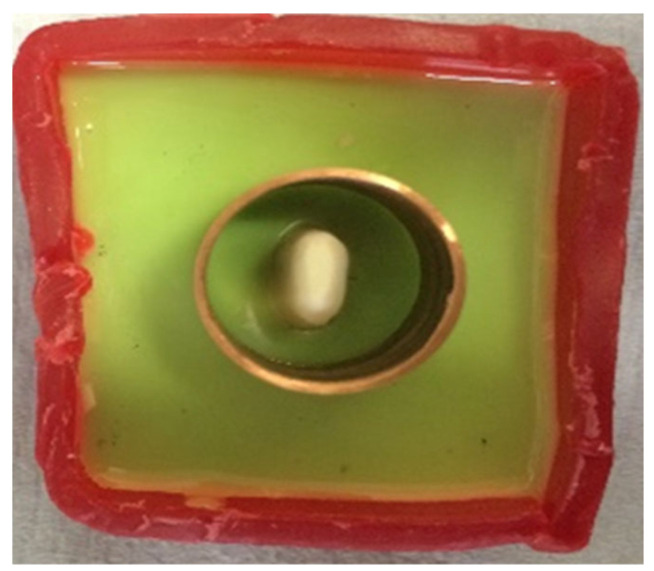
A copper ring was placed over the mould to evenly surround the sample root to ensure it was ready to fill with freshly mixed bone-like material AlphaDie^®^ MF.

**Figure 9:**
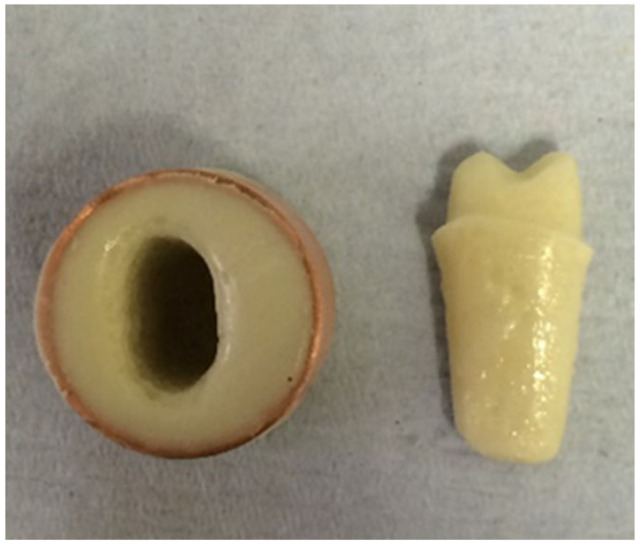
When it had initially set the sample was gently detached from the ring base leaving a space, this hollow simulates the alveolar bone socket which surrounds the natural teeth.

Addition silicon impression material Dublisil^®^ [15] was used to create duplication moulds as in Figure 1, Figure 2 and Figure 3. Replicas of the prepared teeth were obtained using the polyurethane-based resin material, AlphaDie^®^ MF (). AlphaDie^®^MF is used in many studies as dentine and supports bone substitute material despite the structural dissimilarity between the anisotropic tubular dentine and the isotropic polyurethane-based resin material. It aims to minimise the natural tooth’s histological and anatomical variabilities [33,34,35]. All replicas were visually examined for defects using X3.5 magnification loupes. Samples were subsequently stored in a dry environment for at least 24 h and the following steps regarding the construction and fabrication of the replica were undertaken, as shown in Figure 4, Figure 5, Figure 6, Figure 7, Figure 8 and Figure 9. 

### 2.3. Periodontal Ligament (PDL) Simulation

**Figure 10:**
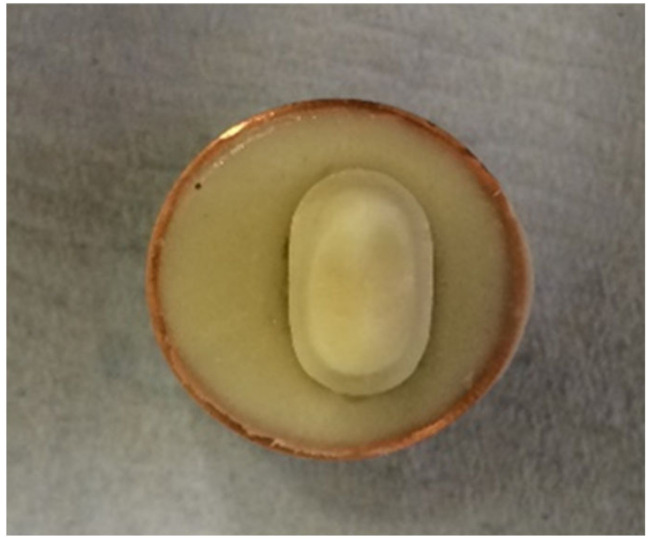
Addition light body silicon impression material (President Plus^®^) injected and reinserted to simulate periodontal ligament.

Previous studies using static and dynamic fatigue testing have demonstrated the need to simulate the periodontal ligament [29,32]. This should resemble the natural physiological grade tooth mobility 0 and allow 50 µm movement matching the natural PDL resilience [20,21,29]. To simulate the periodontal ligaments, a 200 µm uniform thickness with the addition of light body silicon impression material (President Plus^®^, Coltène, Altstätten, Switzerland) was injected around the root. It was injected slowly into the socket then the tooth was immediately reinserted in its position after the injection as depicted in Figure 10. The tooth was then confirmed to exhibit no physiologic tooth mobility (Grade 0) as per normal tooth mobility. Excess injection material was removed using size 15 and 11 surgical blades.

### 2.4. Crown Fabrication

Crowns were fabricated on the master Frasaco tooth preparations. The InEos (inEos x5, Dentsply Sirona, Charlotte, NC, USA) system and CEREC inLab (Version 3.60) (VITA, Zahnfabric, Germany) were used for scanning and designing the crowns. Each milled crown was carefully examined using magnification (3.5×). Any defective crowns were discarded, and the remaining were tested. Satisfactory marginal and base adaptation and correct occlusal surface morphology were required before the crowns were deemed adequate for cementation. 

### 2.5. Crown Cementation

The teeth were cleaned with a rubber cup and pumice using a low-speed handpiece. The crowns were cemented using material recommended by the manufacturer which was used in accordance with the prescribed instructions [31,32,36,37]. The cementation steps and agent used are highlighted below in Table 2.

#### Standardised Cementation Techniques

**Figure 11:**
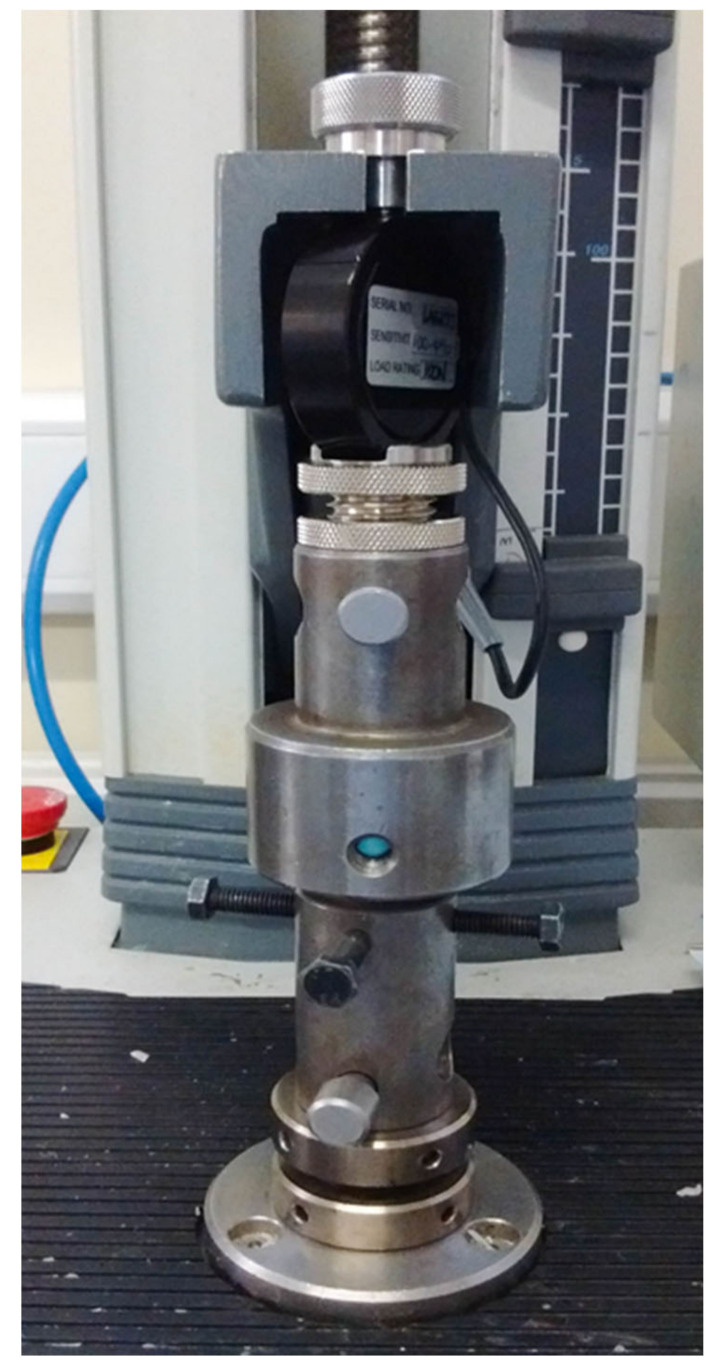
Final cementation settings by using the tensometer.

A standardised cementation procedure was applied to ensure an adequate and consistent cementation force in order to achieve a uniform thickness of luting cement. A custom-made device attached to a tensometer (Lloyds Instrument Model LRX, Hampshire, UK) was used to deliver a constant force of 40 N over 3 min to simulate the finger pressure and equal distribution of force throughout the occlusal surface [32,35,38]. A tested and calibrated visible blue light (LED) light-curing unit was used to ensure complete polymerisation at the margins of the crown (Figure 11).

### 2.6. Finishing and Polishing of the Samples

Residual excess cement was removed with a plastic hand instrument. A standard procedure of cleaning and polishing the crowns and margins was used for all samples using the following sequence: brown and green silicone rubber points (Shofu Inc., Kyoto, Japan) and Sof-Lex™ finishing and polishing discs (3M™ ESPE, St Paul, MN, USA) in a low-speed handpiece. All the samples were left undisturbed for at least 1 h and kept immersed in a water solution at a constant of 37 °C for at least 24 h before testing. 

### 2.7. Mechanical Testing

#### 2.7.1. Static Testing

**Figure 12:**
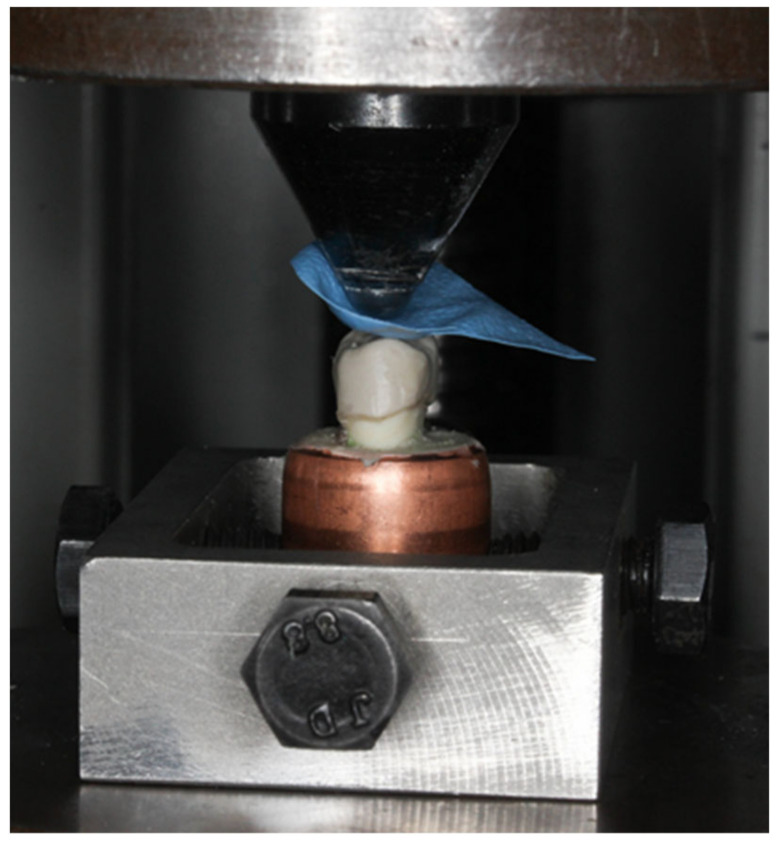
Marks were made initially on the inclination of both the buccal and palatal cusp.

**Figure 13:**
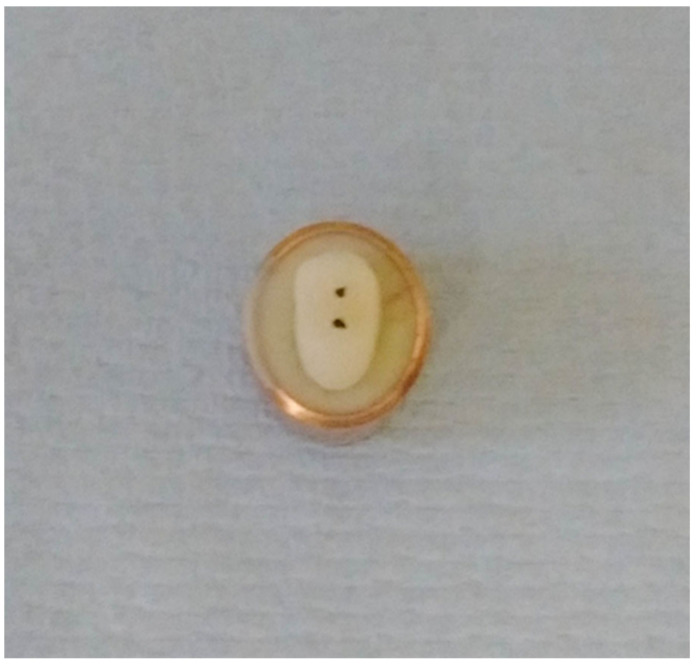
Sample set-up prior to testing.

A universal testing machine (Lloyds Instrument Model LRX) was used for this procedure. The teeth were mounted to the fixed base of the machine parallel to the crosshead. A crosshead with a speed of 1 mm/min with a standard diameter ball head (4.24 mm) was used as a static compression axial load indenter [14,15] (Figure 12). A rubber dam (2 × 2 cm) with a standard thickness of 1 mm was used as a stress breaker (Figure 13). The maximum compression force was recorded in Newton (N) [32,38]. 

#### 2.7.2. Dynamic Testing

**Figure 14:**
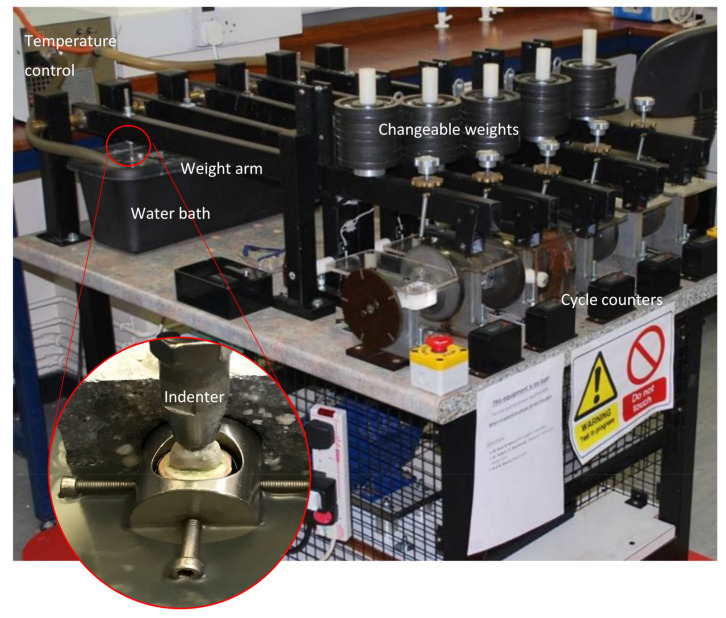
Chewing simulator machine.

The specimens were mounted in the chewing simulator machine which has been designed and constructed by Professor Mair for a study assessing the fatigue limit and fatigue life in resin-bonded metal to enamel bonds [15,33] (Figure 14). It works by applying a repetitive axillary changeable load on the mounted specimens in a water bath with five fixed sample stations linked with a water temperature regulator made to simulate the intraoral environment. Variable movable metal discs were placed at the end of the five individual loading arms to generate and control the load magnification delivered on the samples at the other end by the indenters. In this test, we mounted the samples at 0° degree in a 37 °C water bath to be subjected to the adjustable spherical tip stainless-steel indenters (4.25 mm tip diameter with silicon cushion disc) attached to the other end of the arms. The uniaxial load direction arms holding indenters at their ends adjusted to occlude on two contact points on each crown, a lingual inclination of buccal cusp and buccal inclination of lingual cusp, these points were evaluated by a thin articulating paper [33]. Light body silicon cushions (President Plus^®^) were placed between the loading arms and the indenters to act as the antagonist teeth PDL, reducing and absorbing the load effect. The load frequency was set at 1 Hertz, meaning one occluding cycle every second. 

##### Fatigue Limit Experiments

A staircase technique was chosen to determine the fatigue limits of the PEEK and IPS e.max^®^CAD crowns individually. This method started by loading the first fresh sample of each group with 453 N occlusal force (4.0 Kg) for 5000 cycles at 1 Hertz cyclic frequency. When the 5000 cycles were completed or the crown fractured, the sample was removed and replaced with a new sample with a 0.5 kg increase of the loading disc (if it survived the 5000 cycles) or a decrease of the loading disc (if it failed prematurely). The fatigue limit was clearly identified when the samples survived three times under the same load after the 5000 cycles and sequentially fractured three times under the next higher load. Subsequently, the survival load (endurance load) was identified as the fatigue limit utilised to determine the fatigue life. The power calculation of previous studies showed that 10 to 20 samples were sufficient to identify the fatigue limit load for different dental materials. Thus, 20 samples for each group were allocated to be used for the fatigue limit test. 

##### Fatigue Life Experiments

Accordingly, the fatigue limit load of each group was used to determine the fatigue life, which was identified as the number of cycles applied on the crowned teeth until they fractured under the assigned occlusal load. The number of samples used for each group depended on the power calculation assigned to identify if a significant difference was achieved between the control and test groups or not. In fatigue life test, more than one sample could be tested at the same time with different counters. The chewing simulator machine was set to run for 1,250,000 cycles at 1 Hertz cyclic frequency. However, automatic cut-off calculators, which calculate the number of applied cycles, will cut off the cyclic load and stop counting when the sample fractures. 

### 2.8. Mode of Fracture

Burke’s fracture mode analysis (Table 3) has been used extensively in assessing and describing the initial fracture pattern of extra-coronal prosthesis tested by in vitro study. It is due to its simplicity, reliability, and practicality for fracture observation and statistical analysis [32,38,39]. The fracture modes of each group and specimens were recorded. 

### 2.9. Fractographic Stereomicroscope Assessment

**Figure 15:**
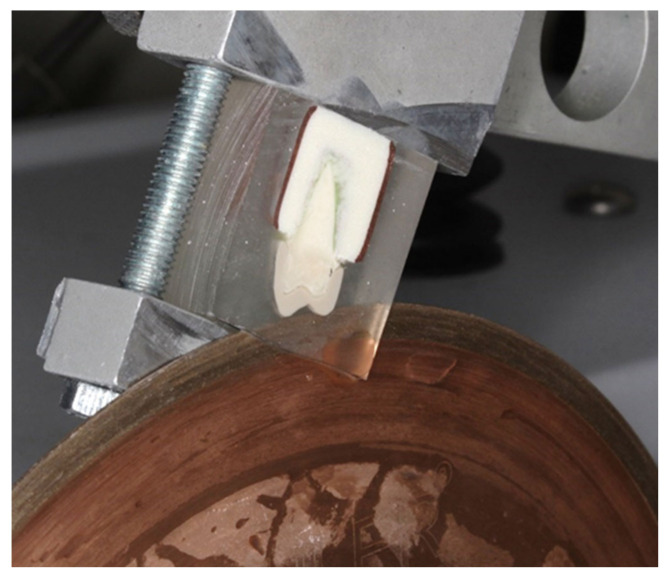
Cold cured acrylic blocks sectioned with diamond discs attached to a cutting machine.

Random samples from the PEEK group were selected for stereomicroscopic analysis. A total of three samples were selected initially and the best samples with the clearest view were chosen as depicted in Figures 19 and 20. The samples were cleaned thoroughly under running water to remove any debris or grease resulting from the testing. They were then sectioned parallel to the long axis of the teeth (Buehler IsoMet^®^ 1000 Precision Saw, Wooster, OH, USA) (Figure 15). The samples were polished (Buehler METASERV^TM^) to create a very fine surface utilising 1200 grit (P1200) finish. 

The samples were examined under a stereomicroscope (ZEISS SteREO Discovery.V8, Medit, Seoul, Korea) under magnification of up to 8× zoom. The samples at the magnification were selected and processed using an imaging software package (AxioVision Rel. 4.8. ZEISS).

### 2.10. Digital Subtraction by 3-D Model Analysis

A 3-dimensional (3D) model analysis was generated from 3 samples chosen randomly after each test and the samples with the best views were selected, as depicted in Figures 22 and 23. They were scanned before the tests and then cleaned thoroughly under running water to remove any debris or grease, followed by optical powder application for scanning CEREC powder VITA. Initially, the samples were then scanned using a 3D scanner (Identica Blue V 1.1.9.5, Medit, Seoul, Korea). A total of four constant points were selected to allow scanning thus enabling comparison from the sample. They were then processed by a finite element analysis (FEA) mesh software for 3D analysis and measurements (3-Matic ^®^Research Version 10.0, Materialise 3-matic, Leuven, Belgium).

## 3. Results

### 3.1. Fracture Strength

The mean fracture strength for both groups was recorded in Newton (N) (Table 4). This was analysed using SPSS statistical package version 21.0. A (*p*) level of 0.05 was used to detect any significant difference (*p* = 0.05). A normality test was done on the groups. Results with *p* > 0.05 mean that the data from the mean fracture strength category are normally distributed. A one way Analysis of Variance (ANOVA) was performed to detect the level of significant difference between groups followed by post-hoc Tukey Pairwise comparisons to analyse which group was the one that demonstrated significant difference. The results show that there was a significant difference in fracture strength between the two groups (Table 4).

### 3.2. Fatigue Limit

**Figure 16:**
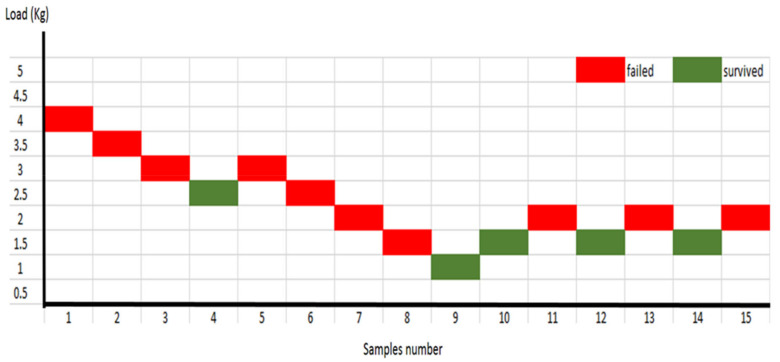
Fatigue limit staircase for IPS e.max^®^CAD crowns.

**Figure 17:**
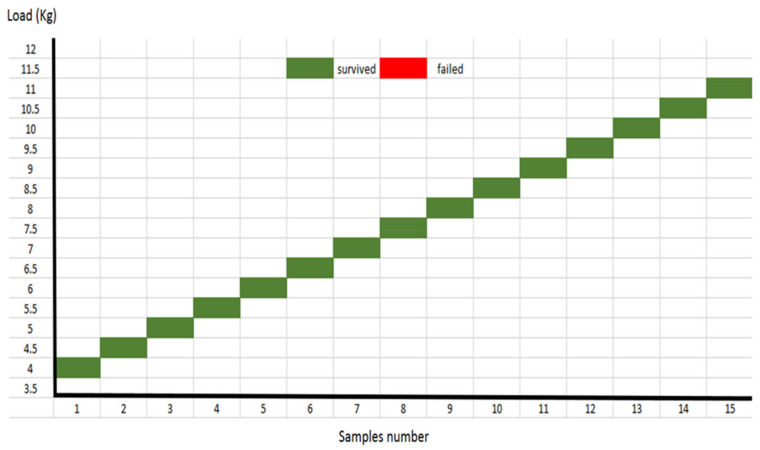
Fatigue limit staircase for PEEK crowns.

IPS e.max^®^CAD crown-tooth samples failed consistently at a load lower than 2.0 kg (=320 N). Meanwhile, at a level of 1.5 kg (=284 N) the samples survived the load for 5000 cycles (Figure 16). Thus, this study selected 1.5 kg (=284 N) as the IPS e.max^®^CAD crowns’ fatigue limit load. PEEK crowns showed continued survival at higher loads. PEEK crown samples did not fracture but showed signs of deformation at higher survival loads of 11 kg (930 N) and potentially more for 5000 cycles (Figure 17). Consequently, an occlusal load that is usually applied on the posterior teeth of 300 N to 500 N [14,28,34,40] was considered more appropriate at 5 kg (522 N).

### 3.3. Fatigue Life

The highest limit of cycles was set to be 1,250,000 representing >5 years of chewing. A total of six samples in each group were subjected to the fatigue life test under the selected fatigue limit (1.5 Kg for IPS e.max^®^CAD crowns, 5.0 Kg for PEEK crowns). The sample size power calculation was 100% for the mean readings of the 12 samples (Source-forge website). The *t*-test statistical analysis illustrates the highly significant difference between the mean values of the survived cycles between the two groups (Table 5). IPS e.max^®^CAD crowns showed early failure after 96,315 to 157,591 cycles (mean = 133,470) (Table 6). However, PEEK crowns survived after 1,250,000 cycles but showed noticeable deformation in the form of dimpling and wear in the crown-indenter contact points which requires further analysis.

### 3.4. Failure Mode Analysis

After a failure in any test, the fracture mode of IPS e.max^®^CAD crowns was assessed according to Burke’s classification, whereas, Kruskal—Wallis analysis was used to classify the fracture mode after the compressive strength static test. It exhibited a significant difference (*p* = 0.000) between the groups (Table 7 and Table 8). In the fatigue testing, the noticeable failure was demonstrated only by the IPS e.max group. The majority of the fracture modes were between code three and five, meaning that half of the crown or more was displaced or lost in code three and four or severe fracture for the crown and tooth in code five (Table 8).

### 3.5. Fractographic Stereomicroscope Analysis

**Figure 18:**
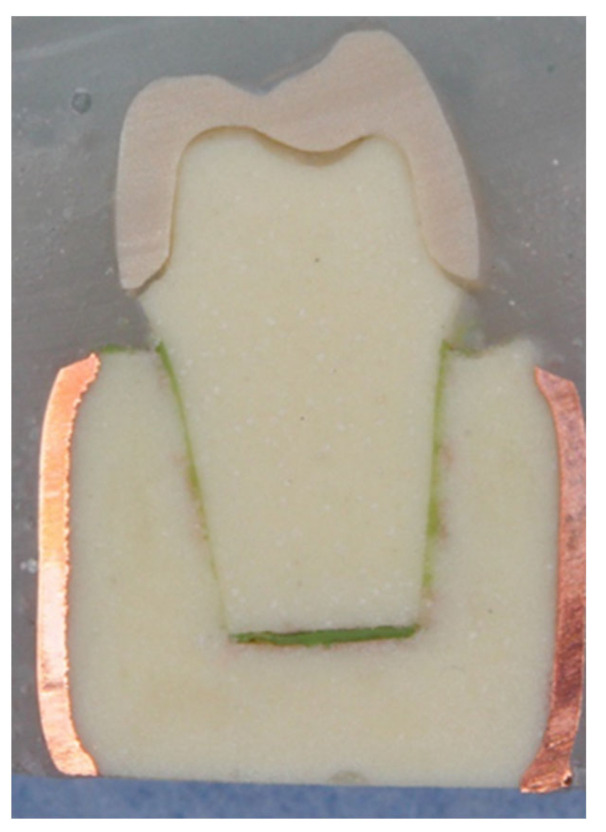
The PDL substitute is the green interface between the tooth structure and surrounding bone replica.

**Figure 19:**
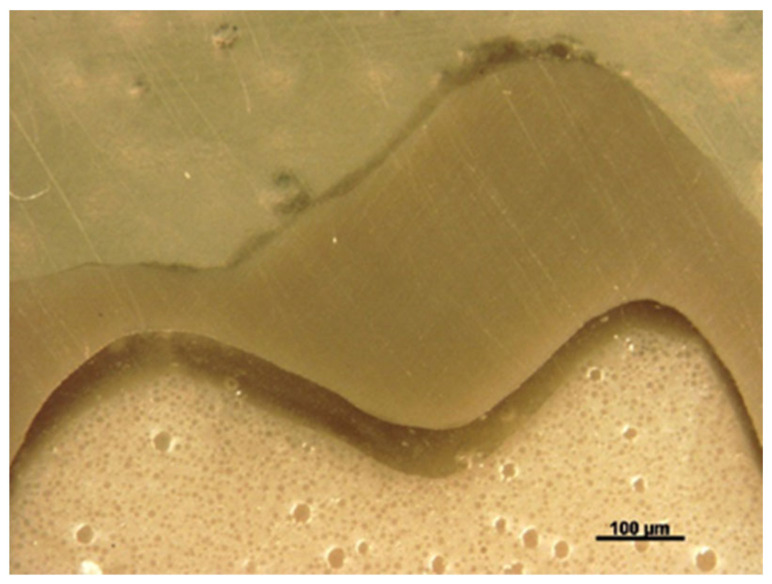
The sectioned photograph of microscopically analysed PEEK crowns before load initiation at 100 µm.

**Figure 20:**
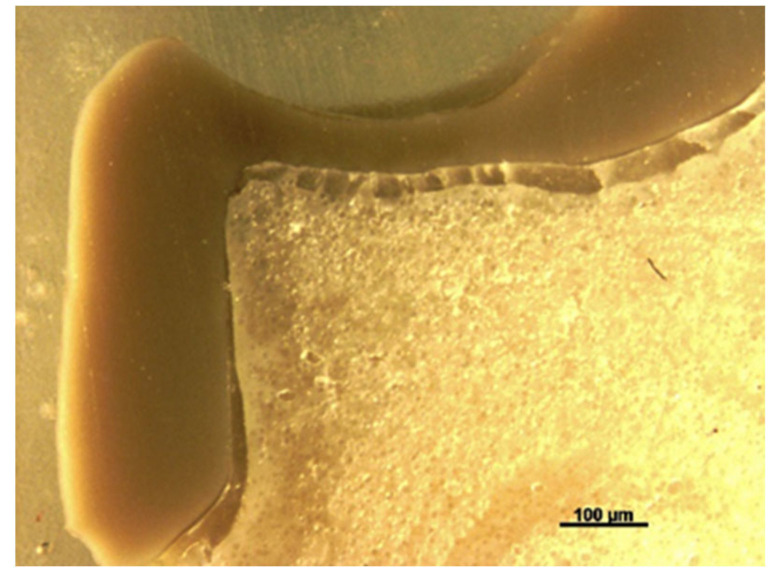
The sectioned photograph of microscopically analysed PEEK crowns after load initiation at 100 µm.

**Figure 21:**
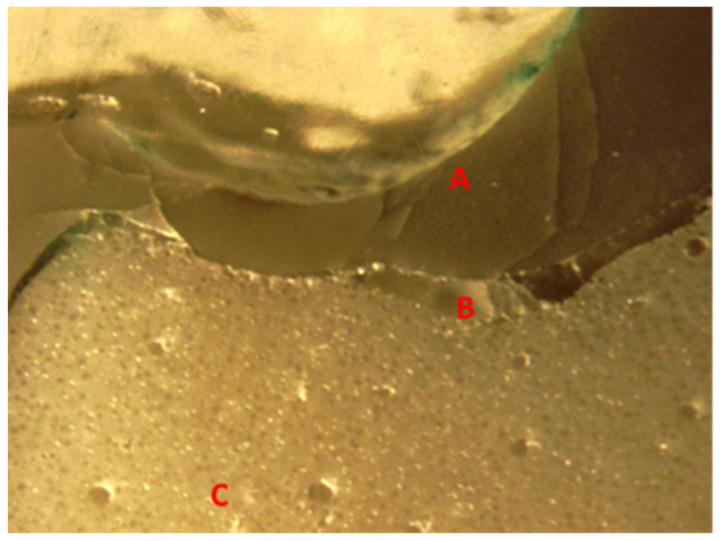
Stereomicroscope image of 1,250,000 cycles fatigue tested (A) damaged PEEK crown (B) crunched and cracked RelyXTM TM Unicem cement interface (C) deformed AlphaDie® MF Polymer tooth.

A cross-section of a cemented non-tested PEEK crown showed a 180–200 µm uniform thickness of simulated PDL, an intact PEEK crown, cement interface within the optimal thickness 10–30 µm and intact underlying polymer AlphaDie^®^MF tooth (Figure 18). The cross-section of the specimen under a stereomicroscope with a magnification of 100 μm is shown in Figure 19. Figure 20 shows the PEEK crown after the compressive test. After the dynamic tests, the PEEK crowns looked physically intact but with compressive plastic destruction of the crown, cement interface and supporting dentine polymer replica (Figure 21).

### 3.6. 3D Digital Subtraction Analysis

**Figure 22:**
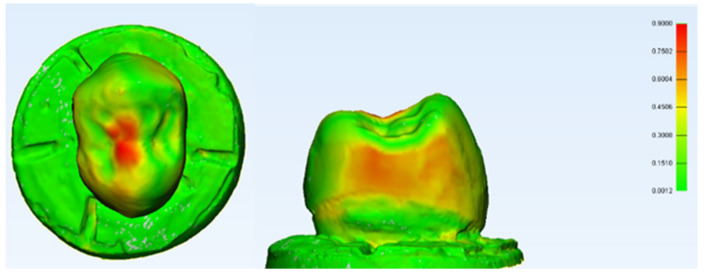
3D submerged pre-and post-test scanning for a PEEK crown after the static compressive test. The technique was colour map-based, as depicted in the above figure. The red represented the most deformed area, the yellow was the lesser deformed area while the green was the area that remained physically unchanged compared to the shape prior to the loading. From the histogram data, the area of deformation happened in the range of 0.0012 mm to 0.90 mm with a mean of 0.1559 mm and a standard deviation (SD) of 0.1586.

**Figure 23:**
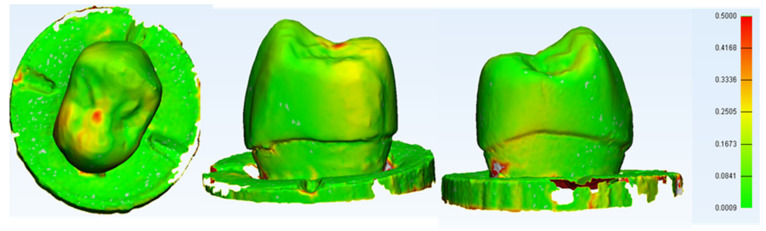
The most visible change was at the contact point between the crown and the indenter which deformed apically by an average of 0.7 mm which represents the overall maximum deformation point. The deformation in the axial walls were generally outward by less than 0.2 mm. The mean of distortion is 0.0913 mm (SD = 0.092).

A 3D model after each test was generated for a non-failed PEEK sample and analysed. The compressive static test result is illustrated in Figure 22 and Figure 23. It shows the PEEK crown sample after undergoing static load compression and dynamic testing, respectively. In dynamic testing, samples were scanned before being subjected to their fatigue limit loads for 5000 cycles and the fatigue life for 1,250,000 cycles. IPS e.max^®^CAD survived crowns did not show any noticeable changes, distortion, or wear. However, PEEK crowns showed some slight noticeable deformation at many walls as depicted in Figure 23. 

## 4. Discussion

Multiple techniques have been utilised in this in vitro study to mimic the situation close to real clinical application. AlphaDie^®^MF was used in this study as it is closely matches the dentin elastic modules (14,800 MPa). This technique can bond to composite luting cement and it can be identically duplicated after the preparation of the intended sample number, which also significantly minimises the natural teeth variables and the variety in the tooth preparations [30,31]. In addition, 200 µm uniform thickness of President Plus^®^ will resemble the natural physiological grade zero tooth mobility and allow 50 µm movement to simulate the clinical presence of periodontal ligament, which has been illustrated in Figure 10 [20]. Resin cement (Rely X Unicem, 3M™ ESPE, St. Paul, MN, USA) is a self-adhesive resin cement with a modulus of elasticity of 8 GPa with the aim of achieving micromechanical and/or chemical bond to the tooth structure. It was used in this study due to its clinical handling simplicity, less technique sensitivity, and is suitable for both types of ceramic and polymer-based crowns [31,36]. Furthermore, the selection of premolar tooth in this study is due to the easiness to detect the contact point during testing and it is the conventionally used tooth selected for in vitro study [41,42]. Milled CAD/CAM PEEK crowns were selected as the test group due to their higher physical and mechanical properties of CAD/CAM PEEK crowns and bridge compared to pressed granular or pellets PEEK [38,43]. Machined lithium disilicate was chosen as a tested group due to similarity in its fabrication and manufacturing methodologies [6]. It also has comparable preparation requirements in both manufacturers’ guidelines. Due to the novelty of PEEK material as a crown, the manufacturers’ guidelines for crown preparation were lacking. Thus, to control the variation, detailed preparation guidelines for e.max crowns were applied to both crowns except the design of the finish line.

The null hypothesis for this study was rejected as the PEEK crown showed a statistically significant difference in the mean fracture strength compared to the control group. The results of this study suggest that the novel PEEK crowns had higher fracture load by using a static test in vitro compared to the monolithic e.max CAD. This was mostly due to the material nature in which it consisted of a thermoplastic polymer chain which allowed plastic deformation to occur under mechanical loading. The maximum fracture load by the PEEK crown was significantly higher than normal mastication with a mean load of 2060.5 Newton (±SD 250.9 N) compared to normal masticatory forces on posterior premolar teeth which can be up to 300 N [44]. These positive findings greatly exceed the normal mastication force on posterior teeth which amounts to around 300–500 N. Nonetheless, the influence of other aspects such as variable preparation design and the effect of the thickness and type of luting cement used were not assessed in this study and it is important to assess the influence of these parameters in a future study.

Dynamic cyclic testing was intended to evaluate a material under interchangeable conditions. For dental material, the dynamic fatigue test is expected to simulate the oral clinical conditions and to preliminarily test the dental material’s clinical performance utilising an in vitro modelling laboratory study. The accumulative fatigue stress had shown to be the most common reason for dental prosthesis failure [24,25]. Thus, it seems logical to investigate the probability of the crown failure from that aspect. In this study PEEK crowns, have been evaluated under fatigue testing, which is designed to simulate to some extent the actual chewing mechanism, which will obtain higher reliability of predicting the long-term survival of the dental crown [35,44,45,46,47]. The nature of PEEK material as a polymer-based material with higher elasticity with a modulus of elasticity of 3.6 Gpa is the reason PEEK crowns can absorb a very high occlusal load shock and survive significantly longer than lithium disilicate ceramic crowns (Modulus of elasticity 95 ± 5 GPa).

Furthermore, the digital subtraction analysis showed that the PEEK crowns deformed in different directions and may be worn in the contact point to compensate for the load applied. In the meantime, the survived ceramic lithium disilicate crowns did not show any deformation before fracture due to their brittleness and disability to deform without fracturing. In fatigue life testing, the data were not consistent within the lithium disilicate group (intra-group variation). Still, the difference between groups (inter-group variation) is significant and this outweighed the large intra-group variation mentioned above. Hence, given the substantial intra-group variation, it is reasonable to accept the data from this investigation based on six samples for each group.

Fractographic analysis provides a further structural analysis of fractography in prosthetic dentistry under the stereomicroscope and scanning electron microscope. Detailed characteristics and effects of different materials within the crown-tooth complex under simulated loading can be assessed. The fracture mode analysis shows that PEEK crowns exhibited significantly higher fracture resistance compared to the lithium disilicate. All specimens of PEEK crowns had shown the capability to withstand higher simulated loading without introducing cracks and fractures on the occlusal surfaces. Conventional ceramics in dentistry generally have low flexure strength; the critical strain of dental ceramics is very low. The material can withstand deformation of approximately 0.1% before fracturing [1,2,48]. Constant repetitive loading and fluctuating stresses and strain may contribute to dental ceramic failure [2]. Furthermore, the presence of crazing and invisible micro-cracks initiated in the surface layer during the crown preparation by the milling machine may quickly be propagated when the load is applied [49]. Although, there is no study, to the author’s knowledge, comparing pressed and milled full-coverage ceramic crowns, Yildiz et al., (2013) have reported that the ceramic onlays fabricated by the pressed technique show higher fracture resistance than ceramic onlays fabricated by CAD CAM technology [35]. Unlike the polymer derived material, the presence of water together with the constant cyclic stress in this test could act as a hydraulic piston which led to an increase in the stress at the crack end of ceramics. It has been found that this combination could result in fatigue failure of the ceramic restoration by up to 50% [15,50].

Fundamentally, the complexity of the tooth crown in this study such as the internal microstructure, composition, and configuration are best analysed under a specific magnification, for example, utilising a stereomicroscope [43]. A sample of a crown tooth complex from a PEEK crown was sectioned, polished adequately and evaluated at the structure level of approximately 100 µm. Prior to the testing, there was a continuous homogeneity on the lower border of the crown, resin cement interface, and underlying tooth structure within the tooth crown complex of the PEEK crown under 100 µm, as depicted in Figure 19. Essentially, a PEEK crown undergoes plastic deformation once it has exceeded its fracture resistant and scores code five on Burke’s fracture mode analysis, which highlights the catastrophic deformation and failure due to this complex tooth-cement-restoration. There was continuous deformation on the margin of the tooth structure, microcracks on the tooth margin and highly compromised structure of the underlying luting cement representing a catastrophic failure of the structural integrity of the samples as depicted in Figure 20 and Figure 21.

A digital subtraction using 3D analysis was made in this study for a random sample of specimens from the PEEK crown group. It showed that a PEEK crown in the sample had coronal and lateral deformation up to 0.9 mm (±SD 0.1586) on the stressed area highlighted by red zonal distribution. The highlighted deformations are also only concentrated on the occlusal and mesial/distal aspects of the crown, highlighting the unpredictable nature of the physical and mechanical properties of the crown under a constant simulated static loading. This was an initial finding; however, it allowed a preliminary estimation of the area that deformed the most and a rough calculation of the deformation area. Nonetheless, further studies are necessary which utilise findings that are better elaborated by using the Finite Element Analysis (FEA) technique, which can accurately measure the stress distribution of the area of interest [51,52,53,54].

This study aimed to mimic, as much as possible, the clinical condition in a simulated oral environment. Nonetheless, there were a few limitations, such as the study only using replica teeth to ensure homogeneity of the samples and one static point selected from the sample as compared to various morphological anatomies of the tooth structures. In future, various testing principles and methodologies such as finite element analysis and clinical trials are very much desirable. The findings of this study showed a potential exciting and additional benefit of using PEEK in terms of mechanical structural integrity strength and durability. These findings further consolidated the usage of PEEK in dentistry [55,56]. In future works, comparison with other available polymers commonly used in dentistry, particularly polymethylmethacrylate (PMMA), is desirable. It might also act as a platform for further investigations on newer high-performance polymers such as PEKK [57,58,59].

## 5. Conclusions

This laboratory-based fatigue testing study can conclude that PEEK is a very promising dental material with desirable mechanical behaviour. It showed greater fracture resistance and in the fatigue limit test, and showed a significantly higher ability to withstand occlusal loads without signs of failure in comparison to lithium disilicate crowns by more than eight times. Monolithic PEEK crowns showed a significantly higher survival rate and significantly less catastrophic failure mode under higher fatigue load tests than clinically successful lithium disilicate crowns in the fatigue life test. In the findings, PEEK crowns seemed to survive in both techniques, the fatigue limit and the life test. However, at a microscopic level, PEEK crowns failed at one point within the 1,250,000 occluding load cycles, and thus need further investigation. Further clinical and laboratory studies are needed to investigate PEEK’s success as a crown material and improve its marginal adaptation and aesthetic properties for PEEK crowns.

## Figures and Tables

**Table 1:** The preparation guidelines as per the manufacturer’s instructions of IPS e.max CAD and PEEK by Juvora^TM^.

Groups	Monolithic Lithium Disilicate IPS e.max CAD	PEEK by Juvora^TM^
Occlusal reduction	1.5 mm	1.5 mm–2.0 mm
Axial reduction	1.5 mm	1.0 mm–1.5 mm
Finish Line	Circular shoulder/chamfer 1.0 mm	Accentuated chamfer 1.0 mm

**Table 2:** List of etchants, bonding agents and luting cement used in the study.

Material	Etching	Silanization	Bonding	Cementation
Monolithic Lithium Disilicate IPS e.max CAD	Hydroflouric Acid Gel 5% (20 s)	Nil	Scotchbond™ Universal Adhesive, 3M ESPE	Rely X Unicem, 3M ESPE
PEEK by Juvora^TM^	Nil	Nil	Scotchbond™ Universal Adhesive, 3M ESPE	Rely X Unicem, 3M ESPE

**Table 3:** Description of different codes of fracture mode as per Burke’s classification 1999.

Mode of Fracture	Description of Each Code
Code 1	Minimal fracture or crack in crown
Code 2	Less than half of crown lost
Code 3	Crown fracture through midline: half of crown displaced or lost
Code 4	More than half of crown lost
Code 5	Severe fracture of tooth and/or crown

**Table 4:** Mean fracture strength, standard deviation, 95% Confidence Interval (CI), and *p* value for each group.

Groups	Monolithic Lithium Disilicate IPS e.max CAD	PEEK by Juvora^TM^
Numbers	10	10
Mean (Newton)	703.4	2060.5
Standard Deviation	47.2	250.9
95% CI	599.7; 807.1	1956.8; 2164.2
*p* value	*p* = 0.0000	

**Table 5:** *t* test comparing the fatigue lives of each group, the mean number of survived cycles, number of samples, standard deviation (SD), T statistics, and *p* value.

Groups	PEEK by Juvora^TM^	Monolithic Lithium Disilicate IPS e.max CAD
Mean	>1,250,000	133,470
Variance	0	4.68 × 10^8^
Number of Samples	6	6
SD	0	21,631.95
T Statistics	126.4301	
*p*-value	<0.0001	

**Table 6:** IPS e.max CAD fatigue life test.

Sample No	Number of Cycles to Fracture
1	147,645
2	96,315
3	157,591
4	140,711
5	122,612
6	135,946
Mean	133,470

**Table 7:** Mode of fracture for each sample within each group after the static compressive testing using Burke’s classification 1999.

Groups	Code 1	Code 2	Code 3	Code 4	Code 5
Monolithic Lithium Disilicate IPS e.max CAD	0	0	4	6	0
PEEK by Juvora^TM^	0	0	0	0	10

**Table 8:** Mode of fracture in IPS e.max CAD after fatigue tests using Burke’s classification 1999.

Test (No of Fractured Samples)	Code 1	Code 2	Code 3	Code 4	Code 5
Fatigue limit test (10)	0	0	2	4	4
Fatigue life test (6)	0	0	0	2	4

## Data Availability

Not applicable.
